# A Spef1-interacting microtubule quartet protein in *Trypanosoma brucei* promotes flagellar inheritance by regulating basal body segregation

**DOI:** 10.1016/j.jbc.2022.102125

**Published:** 2022-06-10

**Authors:** Kieu T.M. Pham, Qing Zhou, Kyu Joon Lee, Ziyin Li

**Affiliations:** Department of Microbiology and Molecular Genetics, McGovern Medical School, University of Texas Health Science Center at Houston, Houston, Texas, USA

**Keywords:** *Trypanosoma brucei*, microtubule quartet, flagellum inheritance, basal body, 3D-SIM, three-dimensional structured illumination microscopy, BSA, bovine serum albumin, FAZ, flagellum attachment zone, FPC, flagellar pocket collar, HA, hemagglutinin, mBB, mature basal body, MtQ, microtubule quartet, NFD, new-flagellum daughter, pBB, probasal body, PLA, proximity ligation assay

## Abstract

The human parasite *Trypanosoma brucei* contains a motile flagellum that determines the plane of cell division, controls cell morphology, and mediates cell–cell communication. During the cell cycle, inheritance of the newly formed flagellum requires its correct positioning toward the posterior of the cell, which depends on the faithful segregation of multiple flagellum-associated cytoskeletal structures including the basal body, the flagellar pocket collar, the flagellum attachment zone, and the hook complex. A specialized group of four microtubules termed the microtubule quartet (MtQ) originates from the basal body and runs through the flagellar pocket collar and the hook complex to extend, along the flagellum attachment zone, toward the anterior of the cell. However, the physiological function of the MtQ is poorly understood, and few MtQ-associated proteins have been identified and functionally characterized. We report here that an MtQ-localized protein named NHL1 interacts with the microtubule-binding protein TbSpef1 and depends on TbSpef1 for its localization to the MtQ. We show that RNAi-mediated knockdown of NHL1 impairs the segregation of flagellum-associated cytoskeletal structures, resulting in mispositioning of the new flagellum. Furthermore, knockdown of NHL1 also causes misplacement of the cell division plane in dividing trypanosome cells, halts cleavage furrow ingression, and inhibits completion of cytokinesis. These findings uncover a crucial role for the MtQ-associated protein NHL1 in regulating basal body segregation to promote flagellar inheritance in *T. brucei*.

*Trypanosoma brucei* is a parasitic protist causing human sleeping sickness in sub-Saharan Africa. This unicellular parasite has a single motile flagellum, which is required for cell motility, cell morphology, and cell division (1, 2, 3). The flagellum is nucleated from the mature basal body (mBB) of the basal body pair composed of a mBB and a probasal body (pBB), with the pBB positioned at the anterior side of the mBB (4, 5). The flagellum exits the cell through the flagellar pocket and further extends toward the tip of the cell anterior. The extracellular portion of the flagellum is attached, for most of its length, to the cell membrane, which is mediated by the flagellum attachment zone (FAZ) (6). During the S-phase, the pBB matures to form a new mBB, and two new pBB’s are formed next to each of the two mBB’s. The newly formed pBB-mBB pair is positioned at the anterior side of the old pBB-mBB pair. A new flagellum is assembled from the newly formed mBB, and the new pBB-mBB pair and the new flagellum then make a rotational move toward the posterior side of the old pBB-mBB pair and the old flagellum (7). Following cell cycle progression, the new flagellum further elongates toward the cell anterior, and upon cell division, two daughter cells are produced, with each daughter cell inheriting a single flagellum.

Faithful positioning and segregation of the newly assembled flagellum is crucial for flagellum attachment, cell division, and cell viability, and it depends on the duplication and/or segregation of several flagellum-associated cytoskeletal structures, including the basal body, the flagellar pocket collar (FPC), the FAZ, and the hook complex (8, 9, 10, 11, 12, 13, 14, 15) (Fig. 1*A*). The hook complex, originally termed the bilobe (16), is a hairpin-like structure composed of two subdomains: a fishhook-like structure marked by TbMORN1 and a bar-shaped structure marked by TbCentrin4 and TbCentrin2 (17, 18, 19). The bar-shaped structure is termed the centrin arm and aligns along the shank part of the fishhook-like structure (17, 18). The hooked part of the fishhook-like structure sits atop the FPC, which is marked by TbBILBO1, and runs alongside the specialized microtubule quartet (MtQ), whereas the shank part of the fishhook-like structure and the centrin arm, which is marked by TbCentrin2 and TbCentrin4, flank the proximal part of the FAZ filament (17). The MtQ is composed of four specialized microtubules (20), which originate between the mBB and the pBB, traverse the FPC, extend in parallel along the intracellular FAZ filament toward the cell anterior, and finally are inserted into the subpellicular microtubule array (21). During the cell cycle, these flagellum-associated cytoskeletal structures are all faithfully duplicated and well segregated into the two daughter cells (Fig. 1*B*), and any defects in their duplication and/or segregation impair flagellum positioning and attachment, inhibit cell division, and eventually lead to cell death.

**Figure 1:**
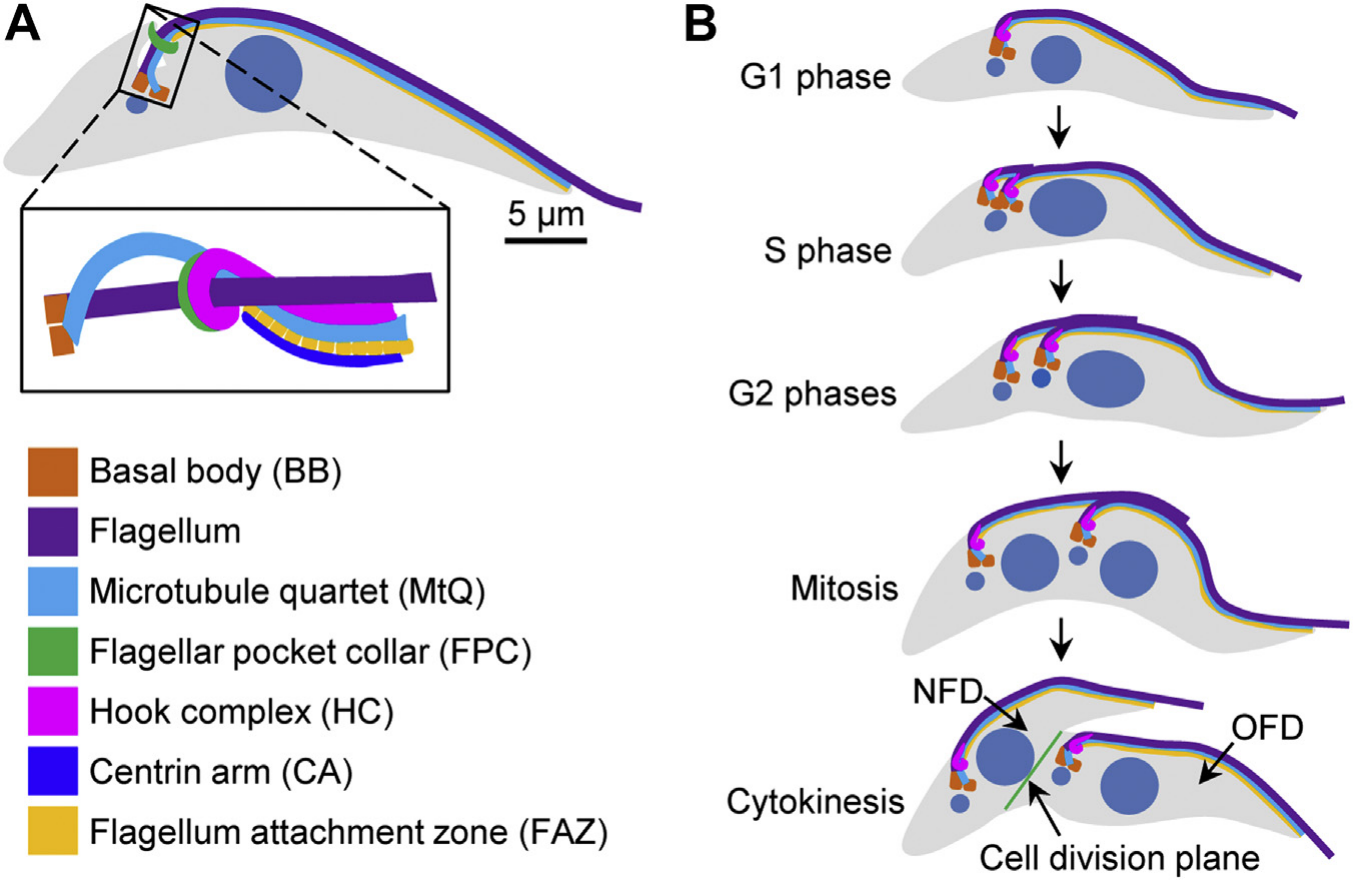
**The organization, duplication, and segregation of flagellum and flagellum-associated cytoskeletal structures during *T. brucei* cell cycle.***A*, schematic drawing of the flagellum and flagellum-associated cytoskeletal structures in *T. brucei*. The scale bar represents 5 μm. *B*, schematic drawing of the duplication and segregation of flagellum and flagellum-associated structures during *T. brucei* cell cycle. NFD, new-flagellum daughter; OFD, old-flagellum daughter.

Cytokinesis in *T. brucei* does not require the conventional actomyosin-based cytokinesis machinery used by its mammalian hosts, and *T. brucei* cells undergo cytokinesis by dividing unidirectionally, along their longitudinal axis, from the anterior tip of the new-flagellum daughter (NFD) cell toward the nascent posterior of the old-flagellum daughter cell (Fig. 1*B*). Prior to cytokinesis initiation, invagination of the plasma membrane occurs between the two flagella, resulting in the formation of the so-called cell division fold (22), which marks the cell division plane. Previous studies reported that the length of the newly assembled flagellum and its associated FAZ correlates with the size of the NFD cell, leading to the proposition that the new flagellum and the new FAZ define the position of the cell division plane (3, 10). However, the underlying mechanisms remain elusive.

Only a few proteins have been localized to the MtQ, of which the *T. brucei* homolog of human sperm flagellar protein 1 (Spef1) was demonstrated as a microtubule-binding protein required for duplication or segregation of several flagellum-associated cytoskeletal structures (23, 24). Here, we report a trypanosome-specific protein named NHL1, which interacts with TbSpef1 at the proximal end of the MtQ. Functional characterization of NHL1 in the procyclic form of *T. brucei* suggests that NHL1 plays essential roles in promoting flagellum-associated cytoskeletal structure segregation, flagellum positioning, and cell division plane placement. These findings uncover an unusual function of NHL1 in facilitating flagellar inheritance in *T. brucei*.

## Results

### NHL1 partly overlays with TbSpef1 at the proximal end of the MtQ

**Figure 2:**
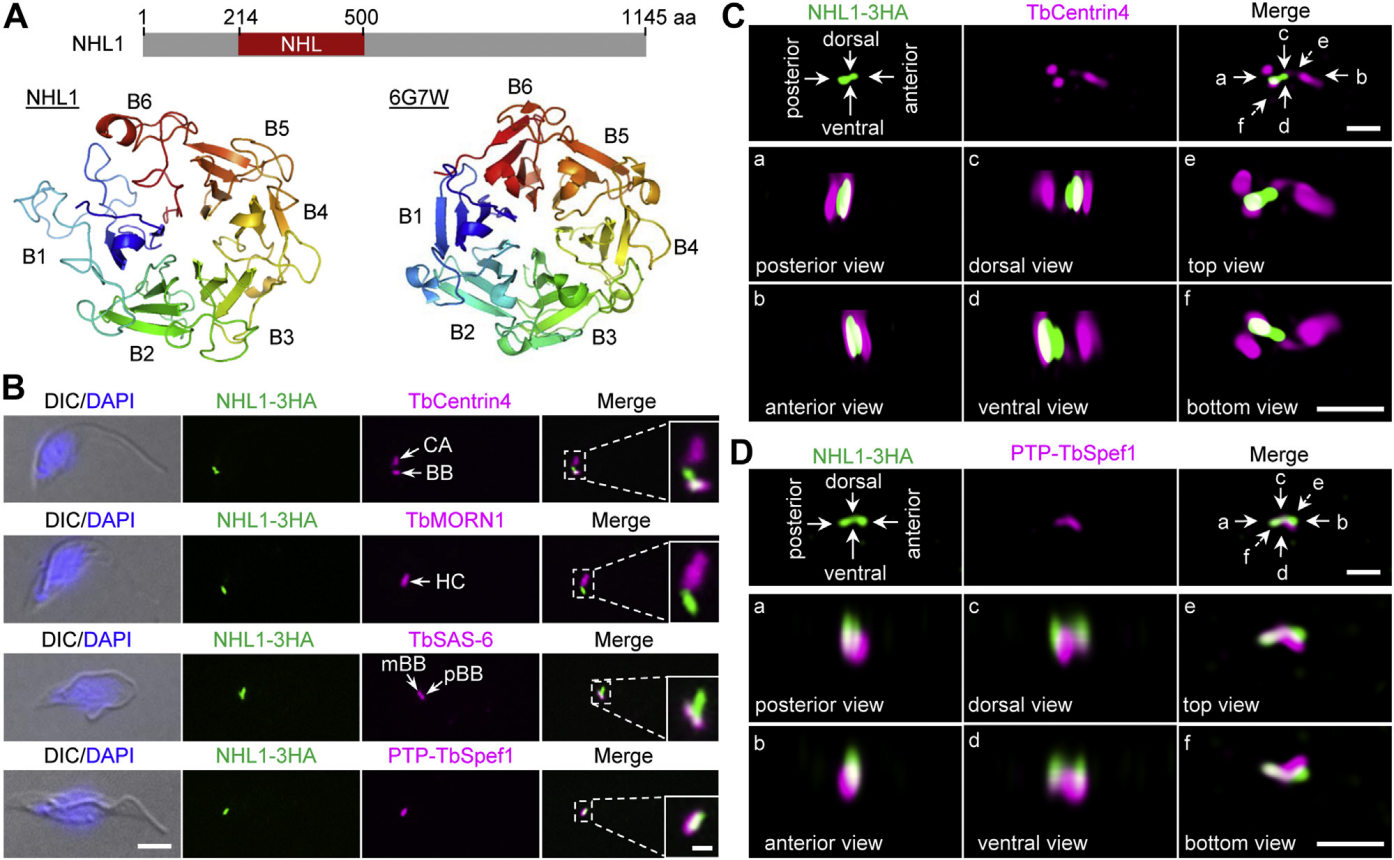
**NHL1 localizes to the proximal portion of the microtubule quartet (MtQ).***A*, schematic drawing of the structural motif in NHL1 (*upper panel*) and homology modeling of the NHL-like domain in NHL1 (*lower panel*) using SWISS model. The template used for modeling was 6G7W. *B*, localization of NHL1 relative to various flagellum-associated cytoskeletal structures by immunofluorescence microscopy. Endogenous NHL1-3HA was detected by FITC-conjugated anti-HA antibody. Anti-TbCentrin4 labels label basal body (BB) and centrin arm (CA), anti-TbMORN1 labels hook complex (HC), and anti-TbSAS-6 labels mature basal body (mBB) and pro-basal body (pBB). Endogenous PTP-TbSpef1 was detected by anti–protein A antibody to label the proximal portion of the MtQ. The scale bar represents 5 μm. The scale bar in the zoom-in image represents 1 μm. *C*, 3D-SIM to examine the localization of NHL1 relative to the basal body and the centrin arm labeled by anti-TbCentrin4. Scale bar: 1 μm. *D*, 3D-SIM to detect the localization of NHL1 relative to TbSpef1. The scale bar represents 1 μm. HA, hemagglutinin.

During the course of work of epitope-based tagging of essential trypanosome proteins for the screening of new FAZ tip-localized cytokinesis regulators (25), a protein encoded by Tb927.9.6160 drew our attention due to its localization to the proximal end of the MtQ (see later). The protein encoded by this gene contains an NHL (Ncl1, HT2A, and Lin-41) repeat-like motif in its N terminus (Fig. 2*A*). Structural modeling using SWISS model (26) showed that the NHL repeat-like motif in this protein appeared to fold into a symmetrical six-blade β-propeller structure, although the blades #1 and #6 were not properly folded, likely due to sequence divergence (Fig. 2*A*). The homologs of this gene are present in *Trypanosoma cruzi* and various *Leishmania* species (www.tritrypDB.org). We named this protein NHL1 and characterized its subcellular localization and cellular functions. Using the cell line expressing NHL1 fused with a triple hemagglutinin (HA) epitope from its endogenous locus, we carried out coimmunofluorescence microscopy with various subcellular structure markers to determine its subcellular localization in the procyclic form of *T. brucei*. Coimmunostaining of cells for NHL1-3HA and TbCentrin4, which marks the basal body and the centrin arm structure of the hook complex, showed that NHL1 localized to a structure connecting the basal body and the centrin arm (Fig. 2*B*). Further, coimmunostaining of NHL1-3HA and TbMORN1, which labels the fishhook-like structure of the hook complex, showed that NHL1 localized adjacent to the proximal end of the hook complex (Fig. 2*B*). Coimmunostaining of NHL1-3HA and TbSAS-6, a basal body cartwheel protein (4), confirmed that NHL1 localized to the anterior of the basal body and showed that NHL1 partly overlaps with the pBB (Fig. 2*B*). These results suggest that NHL1 may localize to the proximal part of the MtQ. To test this possibility, we performed coimmunostaining of NHL1 with TbSpef1, which was previously demonstrated to localize to the proximal part of the MtQ (23), and showed that there was a strong overlap of NHL1 with TbSpef1 (Fig. 2*B*). Using three-dimensional structured illumination microscopy (3D-SIM) super-resolution microscopy, we examined the subcellular localization of NHL1 at a higher resolution and confirmed the localization of NHL1 to the structure connecting the pBB and the hook complex (Fig. 2*C*) and the partial colocalization of NHL1 with TbSpef1 (Fig. 2*D*). Consistent with our results, the TrypTag project also showed that mNeoGreen-tagged NHL1 localizes to the MtQ (27). Taken together, these results demonstrated that NHL1 localized to the proximal part of the MtQ.

### NHL1 is required for completion of cytokinesis

**Figure 3:**
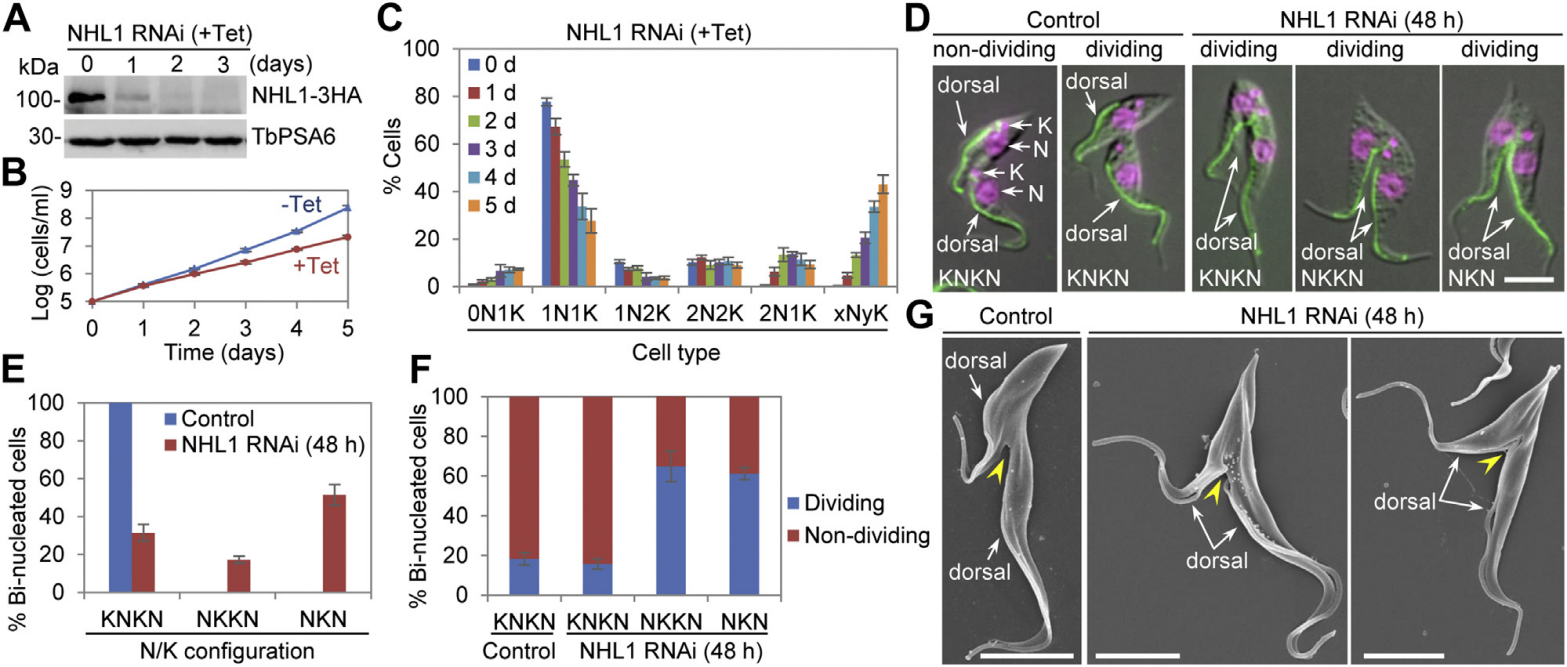
**NHL1 is required for completion of cytokinesis.***A*, RNAi of NHL1 in procyclic trypanosomes. Endogenous NHL1-3HA was detected by anti-HA antibody. TbPSA6 was detected by anti-TbPSA6 as a loading control. *B*, effect of NHL1 RNAi on cell proliferation. Shown is the growth curve of NHL1 RNAi cell line, a representative clone of two clonal RNAi cell lines, which showed similar growth defects. (n = 3). *C*, effect of NHL1 knockdown on cell cycle progression. Shown is the counting of cells with different numbers of nuclei (N) and kinetoplasts (K). Error bars indicate SD (n = 3). *D*, NHL1 depletion caused defects in kinetoplast segregation, cleavage furrow placement, and cytokinesis completion. *Green*: FAZ filament labeled with anti-CC2D antibody; *pink*: nuclear DNA and kinetoplast DNA stained with DAPI. The scale bar represents 5 μm. *E*, effects of NHL1 knockdown on kinetoplast segregation. Shown is the quantitation of binucleated cells with different configuration of the nucleus and the kinetoplast. Error bars indicate SD (n = 3). *F*, quantitation of dividing and nondividing cells. Error bars represent SD (n = 3). *G*, scanning electron microscopic analysis of dividing cells. The *yellow arrowheads* indicate the ingressing cleavage furrow. The scale bar represents 5 μm. DAPI, 4′,6-diamidino-2-phenylindole; FAZ, flagellum attachment zone; HA, hemagglutinin.

To explore the physiological function of NHL1, we carried out a tetracycline-inducible RNAi in procyclic trypanosomes. We endogenously tagged the NHL1 with a C-terminal triple-HA epitope in the NHL1 RNAi cell line for monitoring the level of NHL1. Western blotting using the anti-HA antibody showed that induction of RNAi by tetracycline caused a significant reduction of NHL1 protein level after 24 h and the depletion of NHL1 protein after 48 h (Fig. 3*A*). Knockdown of NHL1 caused moderate growth defects (Fig. 3*B*), demonstrating that NHL1 is important for cell proliferation in procyclic trypanosomes. We next counted the cells with different numbers of nucleus (N) and kinetoplast (K) before and after NHL1 RNAi induction for up to 5 days to examine whether there was any defect in cell cycle progression. Induction of NHL1 RNAi caused a gradual decrease of cells containing one nucleus and one kinetoplast (1N1K) and cells containing one nucleus and two kinetoplast (1N2K), and resulted in the emergence of several abnormal cell types, such as the cells containing two nuclei and one kinetoplast (2N1K), the cells containing only the kinetoplast (0N1K), and the cells containing more than two nuclei and various numbers of the kinetoplasts (xNyK, x > 2; y ≥ 1) (Fig. 3*C*). The emergence of 2N1K cells after NHL1 RNAi was attributable to either the aberrant division of the 2N2K cells, which generated 2N1K and 0N1K cells, or the defective duplication/segregation of the kinetoplast during the cell cycle progression from G1 phase (1N1K cells) to mitosis (2N2K cells). Although the number of 2N2K cells did not change after NHL1 RNAi, the number of binucleated cells (2N2K and 2N1K) was increased (Fig. 3*C*). Moreover, given the emergence and gradual increase of xNyK cells after NHL1 RNAi induction (Fig. 3*C*), these results suggest that NHL1 RNAi caused defective cell division in *T. brucei*.

In WT trypanosomes, all of the binucleated (2N2K) cells have a KNKN configuration (viewed from the cell posterior to the cell anterior), whereas after NHL1 RNAi induction for 48 h, the binucleated cells of the KNKN configuration decreased to ∼32% and the binucleated cells of the NKKN configuration and of the NKN configuration emerged to ∼17% and ∼51%, respectively (Fig. 3, *D* and *E*). In the noninduced control, ∼18% of the KNKN-type bi-nucleated cells were undergoing cytokinesis, and in the RNAi-induced cells, ∼16% of the KNKN-type binucleated cells were undergoing cytokinesis, but ∼65% of the KNNK-type and ~61% of the NKN-type binucleated cells were undergoing cytokinesis, as determined by the presence of a visible cleavage furrow (Fig. 3, *D* and *F*). This result suggests that the completion of cytokinesis in the binucleated cells of the NKKN-type and the NKN-type, but not the KNKN-type, was inhibited. A closer examination of the dividing binucleated cells showed that the two daughter cells were mispositioned in NHL1 RNAi-induced cell population, with the dorsal sides of the two daughter cells being placed next to each other at the cleavage furrow (Fig. 3, *D* and *G*), in contrast to that of the noninduced NHL1 RNAi cell line, in which the dorsal sides of the two daughter cells were far separated and the cleavage furrow was placed between the ventral side of one daughter cell and the dorsal side of the other daughter cell (Fig. 3, *D* and *G*). These results indicate that NHL1 depletion might impair the placement of the cleavage furrow or the positioning of the newly assembled flagellum.

### NHL1 is required for positioning the cell division plane

**Figure 4:**
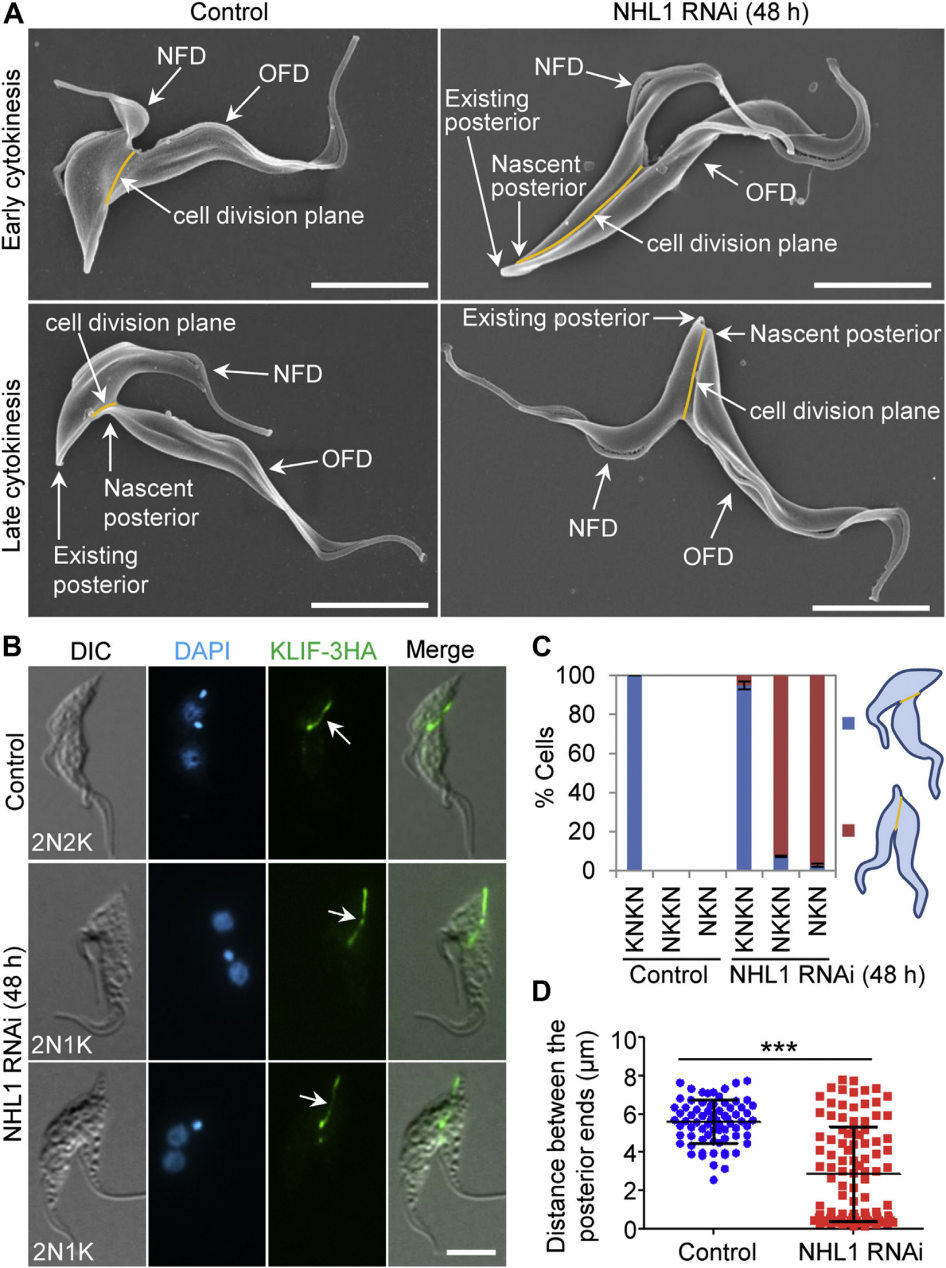
**Depletion of NHL1 disrupts the positioning of the cell division plane.***A*, scanning electron microscopy to examine the cell division plane of dividing cells. The scale bar represents 5 μm. *B*, examination of the cell division plane of dividing cells using KLIF as a marker. Endogenous KLIF-3HA was detected by FITC-conjugated anti-HA antibody. *Arrows* indicate the KLIF-3HA signal that marks the cell division plane. The scale bar represents 5 μm. *C*, quantitation of dividing binucleated cells with normally positioned (*blue*) and mispositioned (*red*) cell division planes. Error bars represent SD (n = 3). *D*, measurement of the distance between the existing posterior and the nascent posterior of the dividing cells from noninduced control and NHL1 RNAi-induced cells. ∗∗∗*p* < 0.001 (Student’s *t* test). HA, hemagglutinin; NFD, new-flagellum daughter; OFD, old-flagellum daughter.

Prior to cleavage furrow ingression, formation of a cell division fold occurs through membrane invagination between the new and the old flagella (22). This division fold marks the cell division plane and lies from the anterior tip of the NFD cell to the nascent posterior of the old-flagellum daughter cell (22, 28). To examine the potential effect of NHL1 knockdown on the formation of the cell division fold and the positioning of the cell division plane, we examined the cells that were undergoing cytokinesis in the noninduced control and the NHL1 RNAi cells using scanning electron microscopy. The noninduced control cells that were at early stages of cytokinesis had a normally positioned cell division plane, with the posterior of the cell division plane (or the nascent posterior) located in the midportion of the NFD cell (Fig. 4*A*). Further, the nascent posterior of the noninduced control cells at late stages of cytokinesis was still connected to the midportion of the NFD cell (Fig. 4*A*). However, the NHL1 RNAi-induced cells that were undergoing cytokinesis had a mispositioned cell division plane, with the posterior end of the cell division plane (or the nascent posterior) located in the proximity of the existing posterior end of the NFD cell (Fig. 4*A*). Using the KLIF protein as a marker of the cell division plane (29, 30), we confirmed that NHL1 RNAi caused mispositioning of the cell division plane (Fig. 4*B*). We further quantitated the percentage of cells that had normally or abnormally positioned cell division plane in the noninduced and NHL1 RNAi-induced binucleated cell population. All the binucleated cells with the KNKN configuration from the noninduced cell population had a normally positioned cell division plane (Fig. 4*C*). In the NHL1 RNAi-induced cell population, ∼95% of the KNKN-type binucleated cells had a normally positioned cell division plane (Fig. 4*C*), but ∼92% of the NKKN-type binucleated cells and ∼97% of the NKN-type binucleated cells had a mispositioned cell division plane (Fig. 4*C*). Finally, we measured the distance between the nascent posterior and the existing posterior of the dividing cells, and the results showed that there was a significant decrease in the average distance from ∼5.8 μm to ∼3 μm after induction of NHL1 RNAi for 48 h (Fig. 4*D*). Altogether, these results suggest that knockdown of NHL1 impaired the placement of the cell division plane.

### NHL1 is required for positioning of the new flagellum

**Figure 5:**
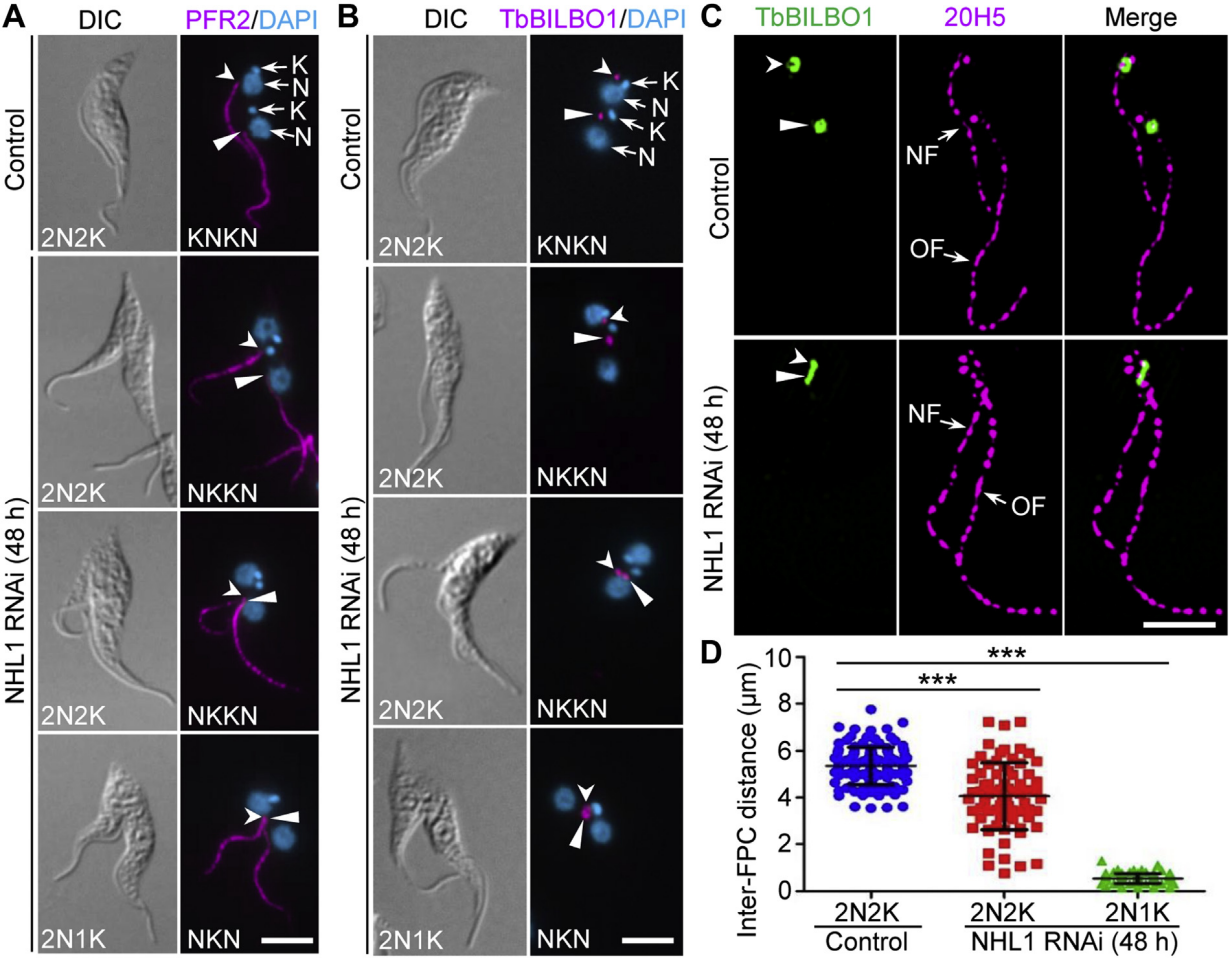
**NHL1 is required for positioning of the new flagellum.***A*, anti-PFR2 immunostaining of the paraflagellar rod (PFR) within the flagella of binucleated cells. *Arrows* and *arrowheads* indicate the proximal ends of the new PFR and the old PFR, respectively. The scale bar represents 5 μm. *B*, anti-TbBILBO1 immunostaining of the flagellar pocket collar (FPC) of binucleated cells. *Arrows* and *arrowheads* indicate the new FPC and the old FPC, respectively. The scale bar represents 5 μm. *C*, 3D-SIM to examine the effect of NHL1 depletion on the segregation of the flagella and the FPCs, which were immunostained with the 20H5 antibody and the anti-TbBILBO1 antibody, respectively. *Arrows* and *arrowheads* indicate the new FPC and the old FPC, respectively. The scale bar represents 1 μm. *D*, measurement of the inter-FPC distance of binucleated cells. ∗∗∗*p* < 0.001 (Student’s *t* test). 3D-SIM, three-dimensional structured illumination microscopy. NF, new flagellum; OF, old flagellum.

Previous studies demonstrated that the length of the new flagellum correlates with the position of the cell division plane in *T. brucei* (3). The finding that NHL1 knockdown affected the positioning of the cell division plane prompted us to investigate whether the positioning of the new flagellum was impaired. We immunostained the noninduced control and NHL1 RNAi-induced cells with the anti-PFR2 antibody, which labels the paraflagellar rod, a lattice-like structure connected to the axoneme in the flagellum (31), as a flagellum marker, and the results showed that the new flagellum in the NKKN-type and NKN-type binucleated cells was not separated from the old flagellum, with the proximal end of the new flagellum sitting in close proximity to the proximal end of the old flagellum (Fig. 5*A*). Further, we immunostained the cells with anti-TbBILBO1 antibody to label the FPC, which showed that the new FPC was not far separated from the old FPC in the NKKN-type and NKN-type binucleated cells after NHL1 RNAi (Fig. 5*B*). Using 3D-SIM, we coimmunostained the cells with anti-TbBILBO1 for FPC and 20H5 for the flagellum, and the data (Fig. 5*C*) confirmed the results obtained with conventional immunofluorescence microscopy (Fig. 5, *A* and *B*). Measurement of the inter-FPC distance in binucleated cells showed that NHL1 knockdown significantly reduced the inter-FPC distance in the NKKN-type and NKN-type binucleated cells (Fig. 5*D*). Together, these results suggest that knockdown of NHL1 inhibited the positioning of the newly assembled flagellum.

### Knockdown of NHL1 disrupts basal body rotation and segregation

**Figure 6:**
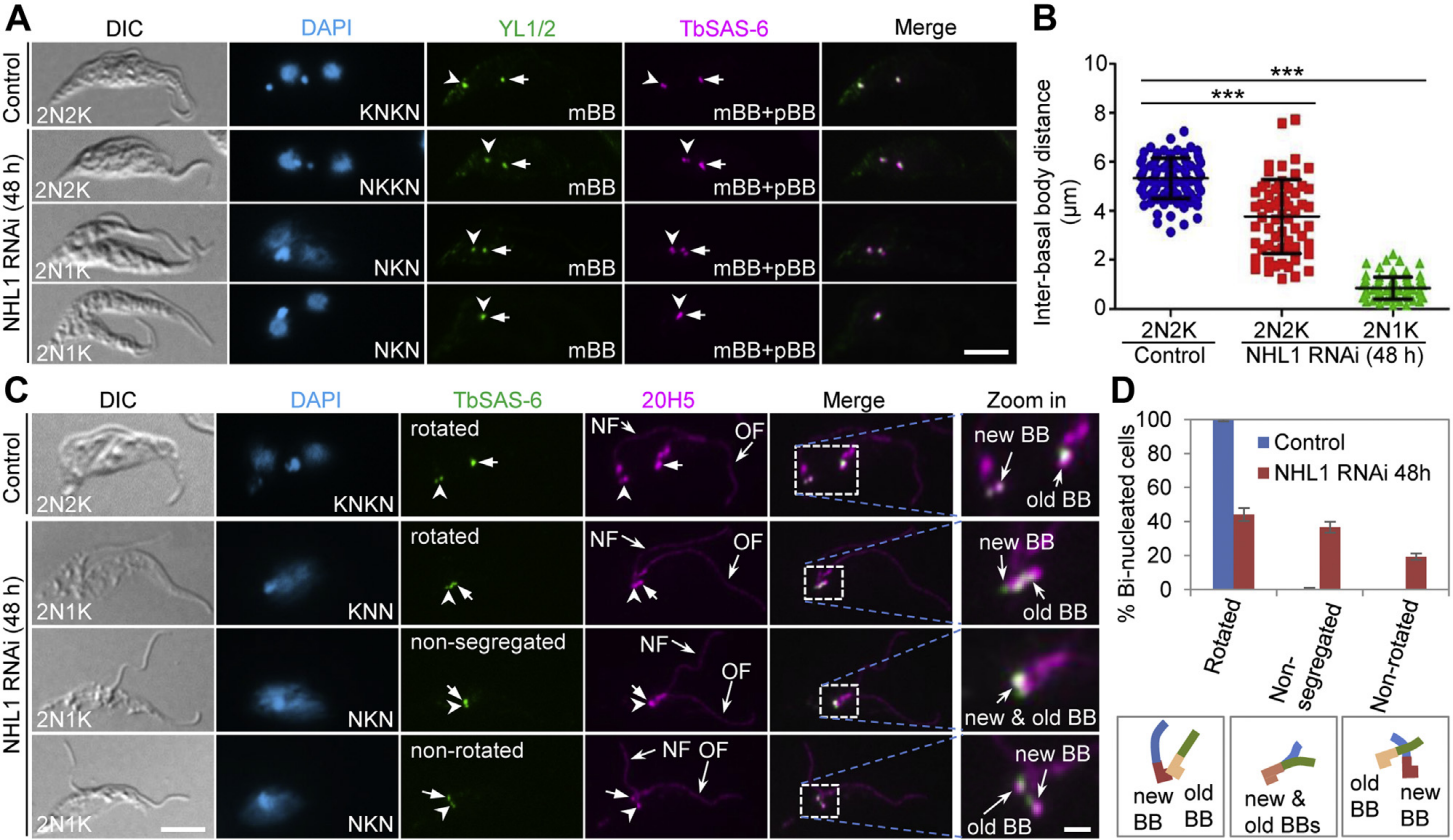
**Depletion of NHL1 disrupts basal body (BB) rotation and segregation.***A*, YL 1/2 and anti-TbSAS-6 immunostaining of the basal body of binucleated cells. *Open arrowheads* and *arrows* indicate new BB pair and old BB pair, respectively. The scale bar represents 5 μm. *B*, measurement of the interbasal body distance of binucleated cells. ∗∗∗*p* < 0.001 (Student’s *t* test). *C*, effect of NHL1 knockdown on basal body rotation and segregation. Anti-TbSAS-6 antibody labels basal body, and 20H5 antibody labels both the basal body and the flagellum. *Open arrowheads* and *arrows* indicate new BB pair and old BB pair, respectively. The scale bar represents 5 μm. The scale bar in the zoom-in image represents 1 μm. *D*, quantitation of binucleated cells with different basal body states. Error bars indicate SD (n = 3). mBB, mature basal body; NF, new flagellum; OF, old flagellum; pBB, probasal body.

During *T. brucei* cell cycle, the new flagellum is assembled from the newly formed mBB, which is accompanied by a newly formed pBB and is located at the anterior side of the old pBB-mBB pair (7). The new pBB-mBB pair further takes a rotational movement from the anterior portion of the old pBB-mBB pair toward the posterior portion of the old pBB-mBB pair and further migrates toward the cell posterior following flagellum elongation and posterior microtubule extension (7). The inhibition of flagellum positioning caused by NHL1 RNAi (Fig. 5) suggests that the segregation and/or the rotation of the newly assembled basal body might also be impaired. We first examined whether basal body segregation was affected by immunostaining the cells with anti-TbSAS-6 antibody, which labels both the mBB and the pBB (4), and YL 1/2 antibody, which labels the mBB (32, 33), and measured the interbasal body distance in the binucleated cells from the noninduced and RNAi-induced cells of the NHL1 RNAi cell line. The results showed that after NHL1 RNAi induction for 48 h, the newly assembled pBB-mBB pair was not far separated from the old pBB-mBB pair (Fig. 6*A*), and the interbasal body distance was reduced from an average of ∼5.8 μm in noninduced cells to an average of ∼3.9 μm in 2N2K cells and ∼0.1 μm in 2N1K cells of the NNHL1 RNAi-induced cell population (Fig. 6*B*). This result suggests that NHL1 knockdown inhibited basal body segregation.

We next investigated whether the rotation of the new pBB-mBB pair was affected by NHL1 RNAi by coimmunostaining the cells with anti-TbSAS-6 antibody for basal body and 20H5 antibody for both the basal body and the flagellum, so that we can distinguish between the newly assembled basal body from the old basal body based on their connected flagellum (Fig. 6*C*). In all the binucleated cells of the noninduced control, the new basal body was found to be placed at the posterior side of the old basal body, indicating that basal body rotation had occurred (Fig. 6, *C* and *D*). In the NHL1 RNAi-induced cell population, ∼44% of the binucleated cells had the new basal body (and its associated new flagellum) placed at the posterior side of the old basal body (and its associated old flagellum) with various distances, indicating that rotation had occurred (Fig. 6, *C* and *D*, rotated), although in many (∼60%) of these cells the segregation of the new basal body from the old basal body was defective (Fig. 6*C*). In ∼19% of the binucleated cells, the new basal body was found to be placed at the anterior side of the old basal body, indicating that rotation had not occurred (Fig. 6, *C* and *D*, nonrotated). In the remaining (∼37%) binucleated cells, the new basal body was found to be too closely associated with the old basal body to determine whether basal body rotation had occurred or not; thus, these cells were assigned to the group of cells with nonsegregated basal body (Fig. 6, *C* and *D*, nonsegregated). Together, these observations suggest that NHL1 depletion inhibited basal body segregation and likely also impaired basal body rotation.

### Depletion of NHL1 impairs the segregation of the hook complex

**Figure 7:**
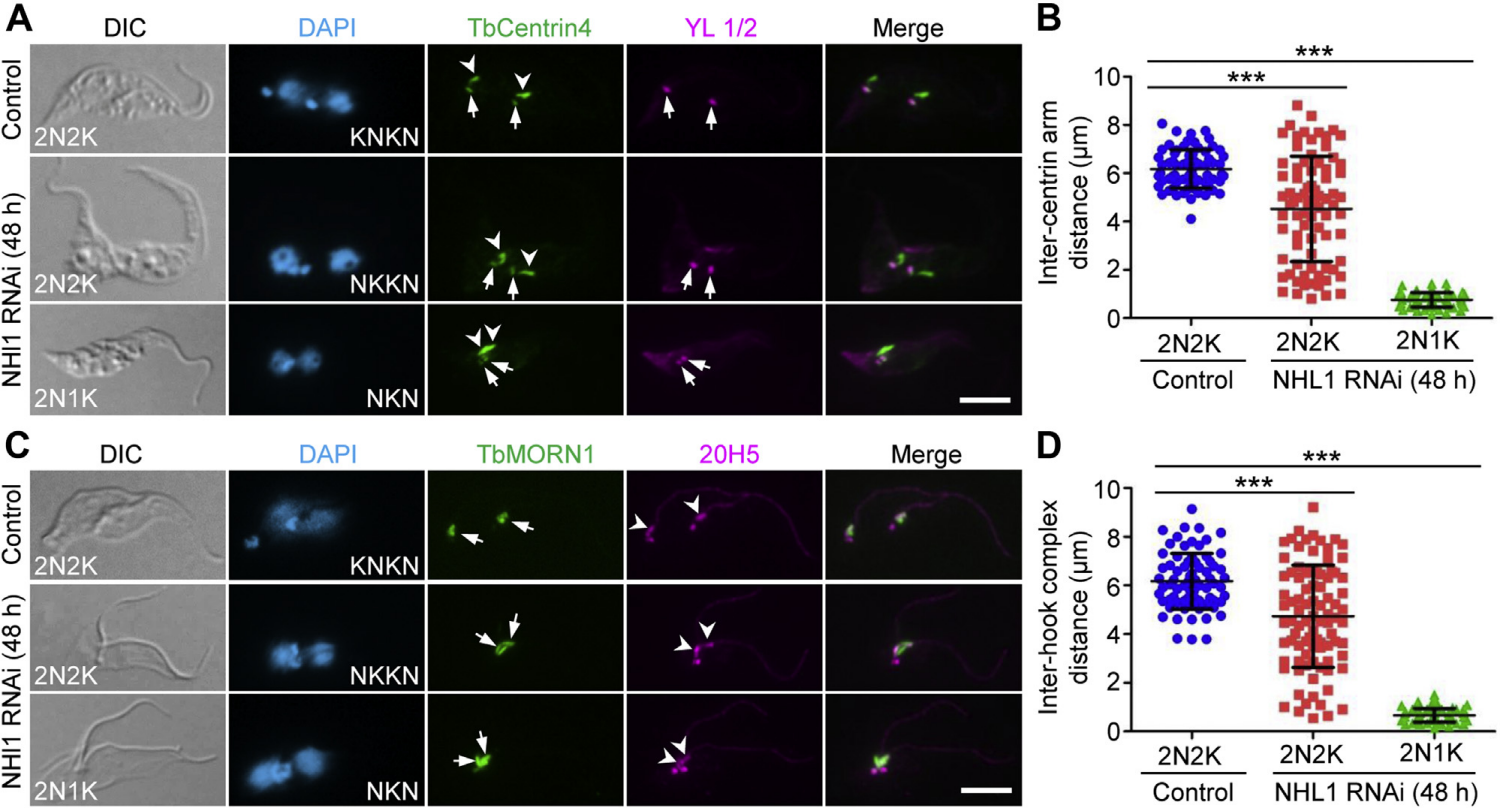
**Knockdown of NHL1 impairs hook complex segregation.***A*, immunofluorescence microscopic analysis of the centrin arm (CA) in binucleated cells. Anti-TbCentrin4/LdCen1 labels the CA and the BB, and YL 1/2 antibody labels the BB. *Open arrowheads* and *arrows* and indicate CA and BB, respectively. The scale bar represents 5 μm. *B*, measurement of inter-CA distance in binucleated cells. ∗∗∗*p* < 0.001 (Student’s *t* test). *C*, immunofluorescence microscopic analysis of the hook complex (HC) in binucleated cells. Anti-TbMORN1 labels the HC, and 20H5 labels the CA and the BB. *Arrows* and *open arrowheads* indicate the HC and the CA, respectively. The scale bar represents 5 μm. *D*, measurement of interhook complex distance in binucleated cells. ∗∗∗*p* < 0.001 (Student’s *t* test). BB, basal body.

We next examined the potential effect of NHL1 knockdown on the duplication and segregation of the hook complex by immunofluorescence microscopy. The hook complex in *T. brucei* consists of a TbMORN1-marked hook complex structure and a TbCentrin2/TbCentrin4–marked centrin arm structure (17). We first examined the centrin arm structure by immunofluorescence microscopy with anti-TbCentrin4 antibody. Since TbCentrin4 additionally labels the basal body, we coimmunostained the cells with YL 1/2 to label the mBB so that we were able to distinguish the TbCentrin4-labeled centrin arm from the TbCentrin4-labeled basal body (Fig. 7*A*). The results showed that after NHL1 RNAi induction for 48 h, the two centrin arm structures were positioned closely to each other in 2N2K cells and were overlapped onto each other in 2N1K cells (Fig. 7*A*). Measurement of the distance between the new and the old centrin arm structures showed that the intercentrin arm distance was significantly decreased in both the 2N2K and 2N1K cells after NHL1 RNAi induction for 48 h (Fig. 7*B*). We next examined the hook complex structure by immunofluorescence microscopy with anti-TbMORN1 antibody, and we coimmunostained cells with 20H5 antibody to label the centrin arm structure (Fig. 7*C*). The results showed that the two TbMORN1-marked hook complex structures were closely positioned in 2N2K cells and were overlapped onto each other in 2N1K cells after NHL1 RNAi induction, similar to the two centrin arm structures (Fig. 7*C*). Measurement of the interhook complex distance confirmed that knockdown of NHL1 significantly reduced the interhook complex distance in both 2N2K and 2N1K cells (Fig. 7*D*). Together, these results suggest that NHL1 is required for segregation of the duplicated hook complexes.

### NHL1 interacts with TbSpef1 and depends on TbSpef1 for localization to the MtQ

**Figure 8:**
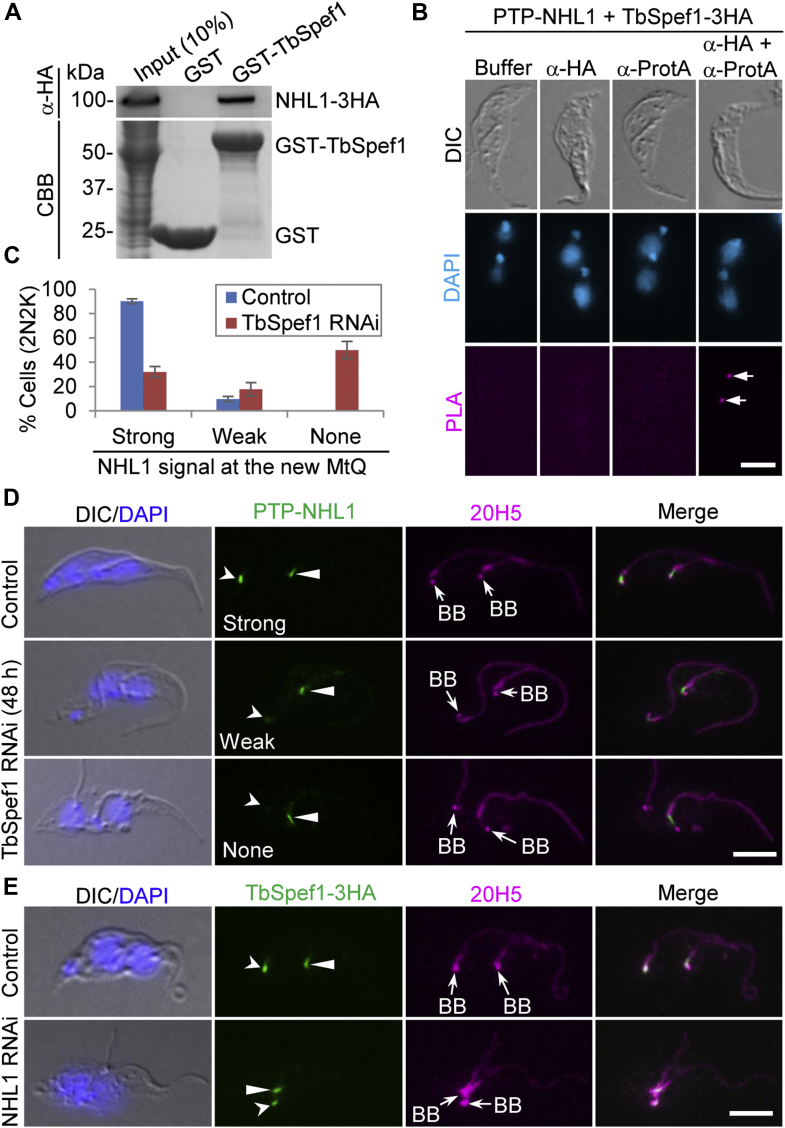
**NHL1 interacts with TbSpef1 and depends on TbSpef1 for localization to the MtQ.***A*, GST pull down to test the *in vitro* interaction between NHL1 and TbSpef1. Shown is a representative of three independent experiments. The input sample was 10% of the ∼475 μl cell lysate used for the pull-down of each bait protein. *B*, proximity ligation assay (PLA) to test the *in vivo* interaction between NHL1 and TbSpef1. Cells coexpressing PTP-NHL1 and TbSpef1-3HA were used. Mock: buffer only. *Arrows* indicate PLA signal. The scale bar represents 5 μm. *C*, quantitation of binucleate cells with different NHL1 fluorescence signal intensity. Error bars indicate SD (n = 3). *D*, effect of TbSpef1 knockdown on NHL1 localization to the MtQ. Endogenous PTP-NHL1 was detected by anti–protein A antibody. *Open arrowheads* and *solid arrowheads* indicate PTP-NHL1 signal in the new MtQ and the old MtQ, respectively. 20H5 was used to label the flagellum, the BB, and the CA. The scale bar represents 5 μm. *E*, effect of NHL1 depletion on the localization of TbSpef1 to the MtQ. Endogenous TbSpef1-3HA was detected with FITC-conjugated anti-HA antibody. *Open arrowheads* and *solid arrowheads* indicate TbSpef1-3HA signal in the new MtQ and the old MtQ, respectively. The scale bar represents 5 μm. BB, basal body; CA, centrin arm; CBB, Coomassie brilliant blue; HA, hemagglutinin; MtQ, microtubule quartet.

The observation of the colocalization of NHL1 with TbSpef1 to the proximal portion of the MtQ (Fig. 2, *B* and *D*) prompted us to investigate whether the two proteins interact or form part of a complex. Using purified recombinant glutathione-*S*-transferase (GST)-TbSpef1 as bait and NHL1-3HA expressed from *T. brucei* as prey, we performed *in vitro* GST pull-down assays, and the results showed that GST-TbSpef1 pulled down NHL1-3HA (Fig. 8*A*). Because TbSpef1 in *T. brucei* was insoluble and thus we could not perform coimmunoprecipitation to test the *in vivo* interaction between TbSpef1 and NHL1, we performed proximity ligation assay (PLA), which detects *in situ* protein–protein interaction in cells with high specificity and sensitivity (34). The PLA method uses two primary antibodies for detecting the two proteins to be examined and two secondary antibodies that are coupled to oligonucleotides (PLA PLUS probe and MINUS probe). If the PLA probes are in close proximity (<40 nm), they are ligated and become amplified by DNA polymerase. Finally, fluorochrome-conjugated oligos hybridize to the amplified DNA, and the PLA signal can be detected by fluorescence microscopy (35). The PLA showed that NHL1 and TbSpef1 were in very close proximity *in vivo* in the proximal region of the MtQ (Fig. 8*B*). Given that NHL1 and TbSpef1 interact, we next asked whether depletion of TbSpef1 affects the localization of NHL1 to the MtQ or *vice versa*. We endogenously expressed PTP-NHL1 in the TbSpef1 RNAi cell line and TbSpef1-3HA in the NHL1 RNAi cell line, respectively, and then performed immunofluorescence microscopy. In TbSpef1 RNAi-induced cells, NHL1 fluorescence signal at the new MtQ, but not the old MtQ, of the binucleated cells became weak or undetectable (Fig. 8, *C* and *D*). Quantitative analysis showed that after TbSpef1 RNAi induction for 48 h, cells with strong NHL1 signal at the new MtQ were reduced by ∼58%, whereas cells with weak NHL1 signal at the new MtQ and undetectable NHL1 signal at the new MtQ was increased by ∼8% and ∼50%, respectively (Fig. 8*C*). Conversely, in NHL1 RNAi-induced cells, TbSpef1 fluorescence signal at the new MtQ of all binucleated cells examined (>1000 cell) was not affected (Fig. 8*E*). These findings suggest that TbSpef1 is required for recruitment of NHL1 to the new MtQ and that TbSpef1 localization to the MtQ is independent of NHL1.

## Discussion

In this article, we identified and functionally characterized a new MtQ-localized protein in *T. brucei*. The MtQ is an unusual flagellum-associated cytoskeletal structure and its molecular function remains poorly defined. Four proteins have previously been localized to the proximal end of the MtQ, of which TbSpef1 functions as a microtubule-binding protein to promote microtubule bundling and coordinate the biogenesis of various flagellum-associated cytoskeletal structures (23, 24). The function of other MtQ-localized proteins and whether any of them forms a complex with TbSpef1 remain unknown. The new MtQ-associated protein identified in this study, named NHL1, partly colocalizes with TbSpef1 at the proximal end of the MtQ (Fig. 2) and interacts with TbSpef1 (Fig. 8). NHL1 contains a divergent NHL-like domain, which is likely involved in protein–protein interaction (36), and does not contain any putative microtubule-binding motifs (Fig. 2). Thus, it is possible that NHL1 is a part of the TbSpef1-containing protein complex that associates with the MtQ through TbSpef1–microtubule interaction.

NHL1 interacts with TbSpef1 at the proximal end of the MtQ, but it appears to play distinct functions from TbSpef1. While knockdown of TbSpef1 impaired the duplication of the MtQ, the FPC, the hook complex, and the FAZ, and inhibited the segregation of the newly assembled basal body and flagellum (23), depletion of NHL1 inhibited the segregation of the flagellum and multiple flagellum-associated cytoskeletal structures (Figure 5, Figure 6, Figure 7). As a consequence of the inhibited assembly of the new FAZ in TbSpef1 RNAi-induced cells, the newly assembled flagellum was detached (23). However, new FAZ assembly was not inhibited in NHL1 RNAi-induced cells (Fig. 3*D*) and, consequently, flagellum detachment was rarely observed in NHL1 RNAi-induced cells. Although TbSpef1 knockdown and NHL1 knockdown both inhibited cell division and produced multinucleated cells, TbSpef1 knockdown inhibited cytokinesis initiation (23) and NHL1 knockdown appeared to block cytokinesis completion (Figure 5, Figure 6, Figure 7 and Figure 5, Figure 6, Figure 7). This distinction in functions on cytokinesis between NHL1 and TbSpef1 likely is correlated with their different roles in regulating FAZ assembly and flagellum attachment. It is known that defective FAZ assembly and, correspondingly, flagellum detachment inhibit cytokinesis initiation (10); thus, the observed defective cytokinesis initiation caused by TbSpef1 RNAi was likely attributed to the defective assembly of the new FAZ. The different phenotypes caused by RNAi of TbSpef1 and RNAi of NHL1 may be attributed to their distinct biochemical functions. TbSpef1 possesses microtubule-bundling activity (24); thus, TbSpef1 may regulate microtubule dynamics in the MtQ, thereby promoting FAZ assembly. However, NHL1 contains only an NHL-like domain, which is potentially involved in protein–protein interaction. Therefore, NHL1 may be a component of a larger protein complex that is recruited to the MtQ (Fig. 8) in a TbSpef1-dependent manner for NHL1 (and possibly TbSpef1’s other interacting partners) to perform functions that are not related to TbSpef1. Finally, given that NHL1 RNAi did not affect TbSpef1 localization to the MtQ (Fig. 8*E*) despite the inhibited segregation of the duplicated flagellum-associated structures caused by NHL1 RNAi (Figure 5, Figure 6, Figure 7), it suggests that TbSpef1 is necessary, but not sufficient, for the segregation of flagellum-associated cytoskeletal structures and that NHL1 needs to be present in order for TbSpef1 to fulfill its roles. Nonetheless, the distinct functions between NHL1 and TbSpef1 suggest diverse physiological roles of MtQ-localized proteins in *T. brucei*.

One of the most prominent phenotypes observed in the NHL1 RNAi-induced cells is the defective segregation of the newly assembled flagellum and multiple flagellum-associated cytoskeletal structures (Figure 5, Figure 6, Figure 7). During trypanosome cell cycle, the newly assembled flagellum and its associated structures are positioned toward the cell posterior, and this process involves the coordination of flagellum elongation and cytoskeletal microtubule extension. The elongation of the new FAZ appears to play a crucial role in controlling the positioning of the new flagellum and the new basal body (10, 11, 37). However, NHL1 RNAi-induced cells were still able to assemble a new FAZ (Fig. 3*D*), albeit the new flagellum and its associated basal body, FPC, and hook complex were not positioned correctly (Figure 5, Figure 6, Figure 7), suggesting that the defect in flagellum positioning was attributed to certain cellular process(es) unrelated to FAZ assembly. The mechanism underlying NHL1-regulated flagellum positioning remains unclear. We speculate that NHL1 might control flagellum positioning through regulating basal body rotation and segregation, based on the finding that NHL1 localizes to the proximal end of the MtQ where it connects the basal body and the hook complex (Fig. 2) and that knockdown of NHL1 inhibits the rotation and segregation of the new basal body (Fig. 6). Because the new flagellum is nucleated from the new basal body, the failure to rotate and segregate the basal body can lead to the failure in positioning the basal body–associated flagellum. The control mechanism for basal body rotation in *T. brucei* remains poorly understood. Because NHL1 localizes to the proximal end of the MtQ and makes contact with the pBB (Fig. 2, *B* and *C*) and that the pBB will develop to form a new mBB from which the new flagellum will be assembled (7), we speculate that NHL1 might play a role to coordinate the rotation of the newly duplicated basal body pair by strengthening the connection between the new basal body pair and the MtQ. When NHL1 is depleted, the weakened connection between the new basal body pair and the MtQ is incapable of pushing the rotational movement of the new basal body pair toward the posterior side of the old basal body pair, thereby causing defective rotation and segregation of the duplicated basal body pairs. A recent study showed that RNAi of the basal body protein TbSAF1 caused the detachment of the MtQ from the basal body, suggesting that TbSAF1 mediates the anchorage of the MtQ to the basal body (24). We have carefully examined the distance between the TbSpef1-labeled MtQ and the basal body in NHL1 RNAi cells, and we found that in all the NHL1 RNAi cells examined, the TbSpef1-labeled MtQ remained to associate with the basal body (Fig. 8*E*). This implies that NHL1 RNAi likely did not cause the detachment of the MtQ from the basal body, unlike TbSAF1 RNAi (24). It would be helpful to examine the structure of the MtQ in NHL1 RNAi cells by electron microscopic analysis of isolated flagella, which will ascertain whether any structural defects occurred on the MtQ by NHL1 RNAi. One possible structural defect of the MtQ in NHL1 RNAi cells is the reduced or lack of bundling of the quartet microtubules at the proximal end of the MtQ, which, as we speculated previously, might weaken the physical connection between the MtQ and the basal body. Future work will be directed to the investigation of the potential effects on the MtQ structure by NHL1 RNAi, the identification of NHL1-interacting proteins, and the potential role of NHL1 in regulating the microtubule-bundling activity of TbSpef1.

An intriguing phenotype caused by NHL1 RNAi is the misplacement of the cell division plane in NHL1-deficient cells that were undergoing cytokinesis (Figure 5, Figure 6, Figure 7 and Figure 5, Figure 6, Figure 7). These NHL1-deficient cells had a mispositioned nascent posterior, which was placed in close proximity to the existing posterior, instead of being placed in the midportion of the dorsal side of the NFD cell as observed in WT cells (Fig. 4). It is known that the cell division plane of a dividing *T. brucei* cell is determined by the length of the new flagellum and its associated FAZ (3, 10). Therefore, the mispositioning of the cell division plane caused by NHL1 RNAi might be correlated with the failure to position the new flagellum toward the cell posterior, although we cannot rule out the possibility that NHL1 plays a direct role in regulating the placement of the cell division plane through a yet unknown mechanism. Nonetheless, the results obtained from RNAi-mediated NHL1 knockdown suggest the requirement of NHL1 for correct placement of the cell division plane.

To sum, we have identified a new MtQ-localized protein named NHL1, which interacts with TbSpef1 at the proximal portion of the MtQ and is recruited by TbSpef1 to the MtQ. Our functional analysis of NHL1 revealed the requirement of NHL1 for segregation of flagellum-associated cytoskeletal structures to ensure flagellum positioning and for placement of the cell division plane to ensure cytokinesis completion.

## Experimental procedures

### Bioinformatics analysis and structural modeling

Analysis of NHL1 structural domains was carried out by the DeepCoil algorithm (https://toolkit.tuebingen.mpg.de/tools/deepcoil2) and hidden Markov models (https://www.ebi.ac.uk/Tools/hmmer/). Homology-based structural modeling of the NHL-like domain in NHL1 was performed using the SWISS model algorithm (https://swissmodel.expasy.org/). The template used for modeling was 6G7W, which is the structure of the NHL domain of the human NHLRC2 protein (38).

### Trypanosome cell culture and RNAi

The procyclic form of *T. brucei* Lister427 strain and 29-13 strain (39) were cultivated in the SDM-79 medium containing 10% heat-inactivated fetal bovine serum (Sigma–Aldrich) and appropriate antibiotics (15 μg/ml G418 and 50 μg/ml hygromycin for the 29–13 strain) at 27 °C, with routine dilutions with fresh medium every 2 to 3 days.

A 875 bp DNA fragment (nucleotides 2363–3237) from the coding region of the *NHL1* gene and a 485 bp DNA fragment (nucleotides 167–651) of the *TbSpef1* gene were each cloned into the pZJM vector (40) to generate the pZJM-NHL1 and pZJM-TbSpef1 plasmids, respectively. The DNA fragment used for NHL1 RNAi was chosen based on its uniqueness (no homologous sequence) in the *T. brucei* genome database. The DNA fragment used for TbSpef1 RNAi has been reported previously (23). Plasmids were linearized and then used for transfection of the 29-13 strain by electroporation. Transfectants were selected in the SDM-79 medium containing 2.5 μg/ml phleomycin and then cloned by limiting dilution in a 96-well plate. Two clonal RNAi cell lines were selected for analysis in three independent experiments. RNAi was induced by incubating the RNAi cell line in the SDM-79 medium containing 1.0 μg/ml tetracycline.

### Epitope tagging of proteins from the endogenous gene locus

We used the PCR-based one-step epitope tagging method (41) to tag NHL1 and TbSpef1 with a C-terminal triple-HA epitope or an N-terminal PTP epitope in *T. brucei*. The PCR products were purified and electroporated into NHL1 RNAi cell line or TbSpef1 RNAi cell line. Transfectants were selected by adding 1 μg/ml puromycin to the culture medium, and cells were cloned by limiting dilution as described previously. Correct replacement of the target genes was confirmed by PCR amplification and subsequent sequencing of the PCR products.

Using the PCR-based epitope tagging method (41), cotagging of NHL1 and TbSpef1 for colocalization was performed by tagging of NHL1 with a C-terminal triple-HA epitope and TbSpef1 with an N-terminal PTP epitope in the Lister427 cell line. For cotagging of NHL1 and TbSpef1 in the same cell line for PLA, NHL1 was tagged with an N-terminal PTP epitope and TbSpef1 was tagged with a C-terminal triple-HA epitope in the Lister427 cell line. Transfectants were selected in the SDM-79 medium containing 10 μg/ml blasticidin and 1.0 μg/ml puromycin, and cells were cloned by limiting dilution as described previously.

### *In vitro* GST pull down

The full-length coding sequence of TbSpef1 was cloned into the pGEX-4T-3 vector (Clontech), and the resulting plasmid and the empty vector were used to transform *Escherichia coli* BL21 strain. Recombinant GST-TbSpef1 protein or GST alone was expressed by induction with 0.1 mg/ml IPTG in 20 ml bacteria culture overnight at room temperature (RT). Bacteria cells were lysed in 1 ml lysis buffer (0.1% Triton X-100 in PBS) by sonication (5 s on and 10 s off for 5 min), and cleared lysate was incubated with 25 μl glutathione sepharose 4B column (GE HealthCare). *T. brucei* cells (∼10^7^) expressing NHL1-3HA were lysed by sonication in 1.0 ml cell lysis buffer (25 mM Tris–HCl, pH 7.6, 150 mM NaCl, 1 mM DTT, 1% NP-40, and protease inhibitor cocktail), and cell lysate was cleared by centrifugation in a microcentrifuge at 4 °C. After centrifugation, 50 μl of the total cell lysate was saved as the input sample, which was mixed with 5 μl 10× SDS sampling buffer. The remaining cell lysate (∼950 μl) was split into two fractions, each of which was incubated with GST-TbSpef1 and GST bound to the glutathione sepharose 4B beads, respectively, for 1 h at 4 °C with gentle rotation. After washing the beads five times with the cell lysis buffer, proteins bound to the beads were eluted by boiling in 30 μl 1× SDS sampling buffer for 5 min. Finally, 15 μl of the eluted proteins and 27.5 μl of the saved input sample were loaded into and separated by SDS-PAGE, and proteins were then transferred onto a polyvinylidene difluoride membrane and analyzed by Western blotting using the anti-HA antibody (1:5000 dilution) to detect NHL1-3HA.

### Immunofluorescence microscopy

*T. brucei* cells were settled on coverslips, fixed with cold methanol at −20 °C, and then incubated in PBS containing 3% bovine serum albumin (BSA). To prepare cytoskeletons for immunofluorescence microscopy, *T. brucei* cells settled on the coverslips were treated with 1% nonidet-P40 in PEME buffer (100 mM PIPEs, pH 6.9, 2 mM EGTA, 0.1 mM EDTA, 1 mM MgSO_4_), fixed with 4% paraformaldehyde, and then blocked with 3% BSA in PBS. Immunostaining was performed by incubating the fixed intact cells or cytoskeletons with the following primary: FITC-conjugated anti-HA mAb (Sigma–Aldrich; 1:400 dilution), anti-TbMORN1 polyclonal antibody (1: 400 dilution) (42), anti-LdCen1/TbCentrin4 polyclonal antibody (1:1000 dilution) (43), anti-TbBILBO1 polyclonal antibody (1:1000 dilution) (44), anti-CC2D polyclonal antibody (1:1000 dilution) (10), anti-TbSAS-6 polyclonal antibody (1:1000 dilution) (4), anti-PFR2 antibody (clone L8C4, 1:100 dilution) (45), 20H5 mAb (1:400 dilution) (46), YL 1/2 mAb (1:1000 dilution) (47), and anti–protein A polyclonal antibody (Sigma–Aldrich, 1:400 dilution). The intact cells or cytoskeletons on the coverslips were washed three times with PBS and then incubated with appropriate secondary antibodies (FITC-conjugated anti-rabbit IgG, Alexa Fluor594-conjugated antirat IgG, Cy3-conjugated anti-rabbit IgG, and Cy3-conjugated antimouse IgG). Finally, cells or cytoskeletons were washed three times with PBS, mounted in the 4′,6-diamidino-2-phenylindole-containing VectaShield mounting medium (Vector Lab), and observed with an inverted fluorescence microscope (Olympus IX71).

### 3D-SIM super-resolution microscopy

3D-SIM super-resolution microscopy was performed essentially as described in our previous publication (48). Briefly, *T. brucei* cells settled on coverslips were treated with 1% NP-40 in the PEME buffer (see previous sections), fixed in cold methanol (−20 °C), and then incubated in PBS containing 1% BSA. Immunostaining was carried out essentially as described previously, except that the cells were viewed under the Nikon Super Resolution Microscope n-SIM E instrument (Nikon Instruments Inc). The acquired SIM images were analyzed by the NIS-Elements AR software (Nikon Instruments Inc).

### PLA

PLA was carried out using the Duolink *In Situ* PLA Detection kit (catalog no.: #DUO92008, Sigma–Aldrich), which includes the Duolink *In Situ* PLA Probe anti-rabbit PLUS (#DUO92002; Sigma–Aldrich) and Duolink *In Situ* PLA Probe anti-mouse MINUS (#DUO92004; Sigma–Aldrich). Briefly, cells were settled on glass coverslips and fix with methanol (−20 °C). Fixed cells were incubated in Duolink blocking solution in a humidity chamber for 60 min at 37 °C and incubated with primary antibodies for 1 h. Cells on the glass coverslips were washed two times with 1× buffer A at RT and then probed with PLUS and MINUS PLA probes for 1 h at 37 °C in a humidity chamber. Cells on the glass coverslips were washed two times with 1× buffer A at RT, incubated with ligation solution for 30 min at 37 °C, and then incubated with the amplification solution in a humidity chamber for 100 min at 37 °C. Finally, cells on the glass coverslips were washed two times with 1× buffer B and one time with 0.01× buffer B, mounted on Duolink *In Situ* Mounting Medium containing 4′,6-diamidino-2-phenylindole, and examined under a fluorescence microscope.

### Scanning electron microscopy

Scanning electron microscopy was performed as previously described (28). Trypanosome cells settled on the glass coverslips were fixed with 2.5% (v/v) glutaraldehyde in dark, dehydrated with a series of alcohol (30%, 50%, 70%, 90%, and 100%), and dried by critical point drying. Using a sputter coater (Cressington Sputter Coated 208 HR, Ted Pella Inc), the glass coverslips were coated with a 8 nm metal film (Pt:Pd 80:20, Ted Pella Inc). Cells were examined using Nova NanoSEM 230 (FEI) with the following parameters settings: 5 mm for the scanning work distance and 8 kV for the accelerating high voltage.

### Data analysis and statistical analysis

We used the ImageJ software (http://imagej.nih.gov/ij/) to measure the distance between the cell posterior ends, between the flagellar pocket collars, between the basal bodies, between the centrin arms, and between the hook complexes. Data were analyzed with the GraphPad Prism5 software (GraphPad Software). Statistical analysis was conducted using the Student’s *t* test, based on the numerical treatments and responses of the experiments (49).

## Data availability

All data are contained within the article.

## Conflict of interest

The authors declare that they have no conflicts of interest with the contents of this article.
